# Genetic-based patient stratification in Alzheimer’s disease

**DOI:** 10.1038/s41598-024-60707-1

**Published:** 2024-04-30

**Authors:** Laura Hernández-Lorenzo, Fernando García-Gutiérrez, Ana Solbas-Casajús, Silvia Corrochano, Jordi A. Matías-Guiu, Jose L. Ayala

**Affiliations:** 1 Department of Computer Architecture and Automation, Computer Science Faculty, Complutense University of Madrid, 28040 Madrid, Spain; 2 Department of Neurology, San Carlos Research Institute (IdSSC), Hospital Clínico San Carlos, 28040 Madrid, Spain; 3 Instituto de Tecnología del Conocimiento, Universidad Complutense de Madrid, 28040 Madrid, Spain

**Keywords:** Machine learning, Alzheimer's disease

## Abstract

Alzheimer's disease (AD) shows a high pathological and symptomatological heterogeneity. To study this heterogeneity, we have developed a patient stratification technique based on one of the most significant risk factors for the development of AD: genetics. We addressed this challenge by including network biology concepts, mapping genetic variants data into a brain-specific protein–protein interaction (PPI) network, and obtaining individualized PPI scores that we then used as input for a clustering technique. We then phenotyped each obtained cluster regarding genetics, sociodemographics, biomarkers, fluorodeoxyglucose-positron emission tomography (FDG-PET) imaging, and neurocognitive assessments. We found three clusters defined mainly by genetic variants found in *MAPT*, *APP*, and *APOE*, considering known variants associated with AD and other neurodegenerative disease genetic architectures. Profiling of these clusters revealed minimal variation in AD symptoms and pathology, suggesting different biological mechanisms may activate the neurodegeneration and pathobiological patterns behind AD and result in similar clinical and pathological presentations, even a shared disease diagnosis. Lastly, our research highlighted *MAPT*, *APP*, and *APOE* as key genes where these genetic distinctions manifest, suggesting them as potential targets for personalized drug development strategies to address each AD subgroup individually.

## Introduction

There are still many unknowns surrounding Alzheimer’s disease (AD), despite decades of study, its high prevalence^1^, and its current and future socioeconomic costs^2–4^. The degenerative, complex^5^, and heterogeneous^6,7^ nature of AD, combined with its action usually in the last years of a person’s life, makes it a silent disease, difficult to detect, and challenging to unravel. These challenges have motivated last years’ research efforts to focus on early AD prediction and development of disease-modifying treatments.

An attractive solution to these two challenges is patient stratification, which consists of identifying subgroups of patients based on sociodemographic, clinical, or molecular data. Identifying these subgroups can help in the development of clinical trials and in the clinical practice itself, helping to provide timely diagnosis and the most appropriate treatment^8,9^ or even refute hypotheses or models of disease functioning^10^. Patient stratification is commonly based on clustering algorithms, which permit discovering new sample groups based on their similarity (or dissimilarity), depending on the definition of the employed clustering algorithm^11^.

In neurodegenerative diseases, clustering applications typically include neuroimaging^12,13^, biomarkers^10,14–16^, neurocognitive data^17^, or even a combination of these data types^18^. However, even though genetics is considered a significant risk factor for the development of AD^4^, machine learning works using genomic data as input are rare, with only a few accounting for genetic patient stratification^19^. Untangling the genetic architecture of AD is key for defining the different pathophysiological profiles, but also in developing a data-driven early and timely diagnosis and providing information on potential targets for new treatments. Moreover, regarding genomics, many recent works have drawn attention to the need to combine genetic data with information about biological networks, such as those involving genes or proteins, as they offer more holistic and accurate solutions regarding the biological processes underlying any disease^20,21^.

In this work, we propose a clustering strategy to derive patients' subgroups based on their genetic variants’ information mapped onto a biological network. Our main objective was to discover new genetic patterns that could explain different pathophysiological or symptomatological profiles within the same disease. With these methodologies, we genetically identified three main profiles and learned about the most affected links in the brain interactome for AD development.

## Results

**Figure 1:**
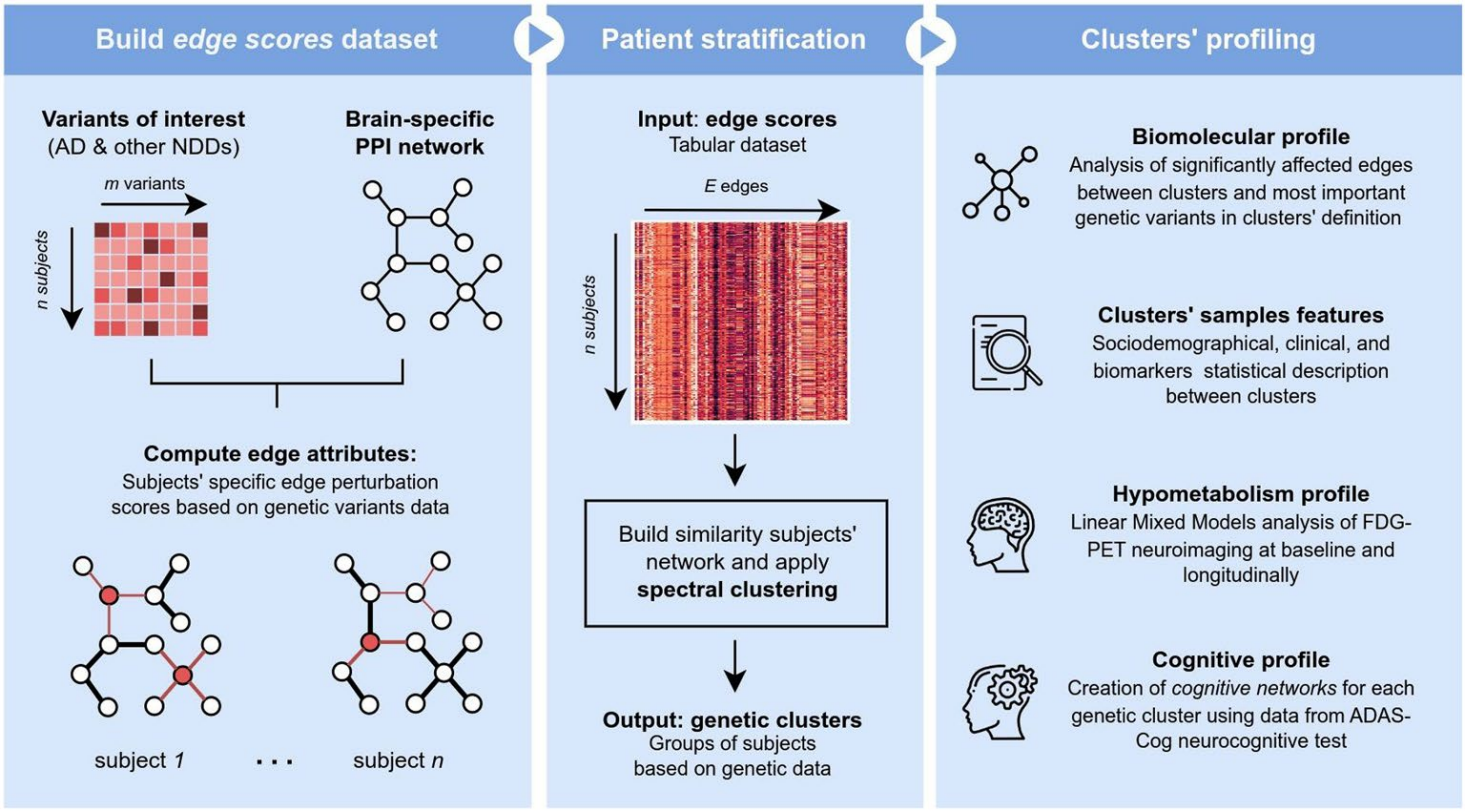
Overview of the presented methodology and results.

In this work, we present a new methodology for patient stratification in an AD cohort according to the effect of their genetic variants on a biological network: a brain-specific PPI. Figure 1 summarizes the developed methodology.

For the development of this work, we employed the Alzheimer's Disease Neuroimaging Initiative (ADNI, adni.loni.usc.edu) genetic cohort. We first selected from this cohort genetic variants found to be previously associated with AD and other neurodegenerative diseases. We then connected the genes in which these variants were found in a brain-specific PPI subnetwork^22^. Next, for each subject, we mapped the variants present in each interacting gene into the PPI’s edges, generating individual edge scores. Thus, each sample resulted with a series of edge scores, representing their altered interactions w.r.t. the original network. We employed these scores as input to a similarity-based clustering model to obtain different genetic profiles of the consulted cohort. Finally, we described each genetic profile from four points of view: (i) genetics, (ii) sociodemographics and common biomarkers, (iii) regional hypometabolism in fluorodeoxyglucose-positron emission tomography (FDG-PET) imaging, and (iv) neurocognitive evaluations.

### Decision on the optimal number of clusters

Any clustering algorithm must evaluate the different model hyperparameters to define the optimal number of clusters for each input dataset, employing metrics such as the Silhouette Index (SI)^23^. In our case, the best solution was two clusters (0.93 SI, Supplementary Fig. 1), followed by a three-cluster outcome (0.75 SI, Supplementary Fig. 1). Both solutions showed a SI value that indicated a very good clustering of the data and maintained a small cluster of 38 samples. Interestingly, the largest cluster in the two-cluster solution was divided into two subgroups in the three-cluster solution. Thus, we decided to explore the three-cluster solution as the most optimal. These three clusters, hereafter referred to as Cluster 1, Cluster 2, and Cluster 3, consisted of 234 (32%), 456 (63%), and 38 (5%) subjects from the original cohort (N = 728). In the following Sections, we will describe each cluster's genetic, sociodemographic, clinical, neuroimaging, and neurocognitive characteristics.

### *MAPT*, *APP,* and *APOE* gene variants mainly define the obtained clusters

**Figure 2:**
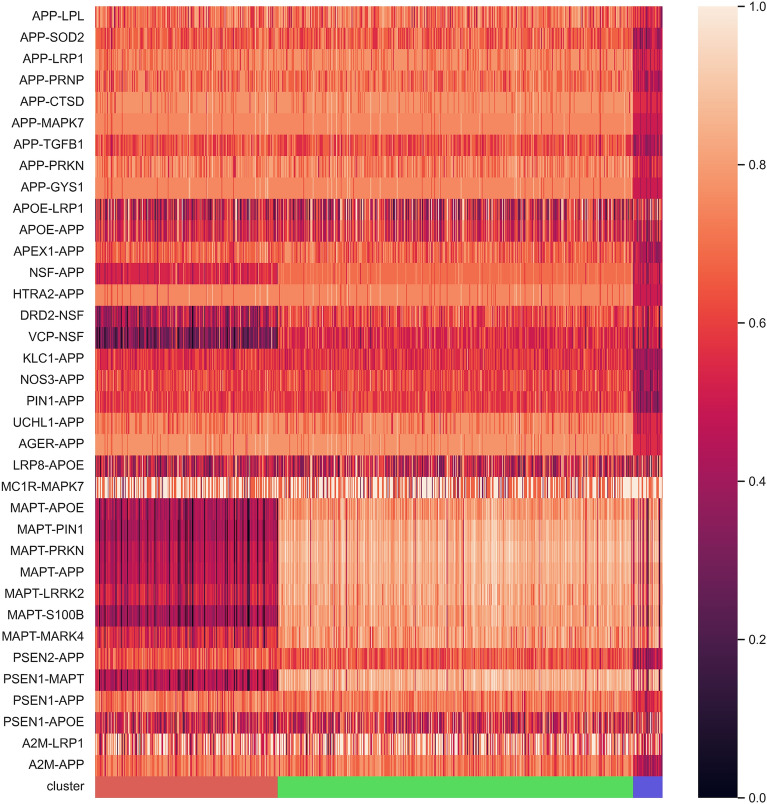
Heatmap showing significantly different edges between clusters. Rows and columns represent gene–gene interaction scores (edge scores) and samples, respectively. Lower values indicate more edge affectation, i.e., more genetic variants in edge interactors. Bottom colors correspond to the obtained clusters: red (Cluster 1), green (Cluster 2), and blue (Cluster 3). Heatmap was generated with seaborn (v.0.12.2)^24^.

Next, we inspected the genetic characteristics that defined each cluster. Figure 2 shows a heatmap of these significantly different scores between the three clusters. Furthermore, Supplementary Table 1 gathers the mean edge scores and the obtained p-values.

The interactions that obtained a significantly different value among the three clusters were those related to *MAPT*. In these cases, the cluster with the most affected *MAPT* interactions was Cluster 1, followed by Cluster 3. On the other hand, interactions with the *APP* gene were significantly affected in Cluster 3. In addition, some slightly less significant differences appeared between *APOE* gene interactions between Clusters 1 and 2, with Cluster 1 having the most affected *APOE* interactions. Finally, we found only one interaction significantly more affected in the case of Cluster 2: MC1R-MAPK7. In general, Cluster 2 was defined through the differences with the other two clusters, being the least affected in all edges overall.

**Figure 3:**
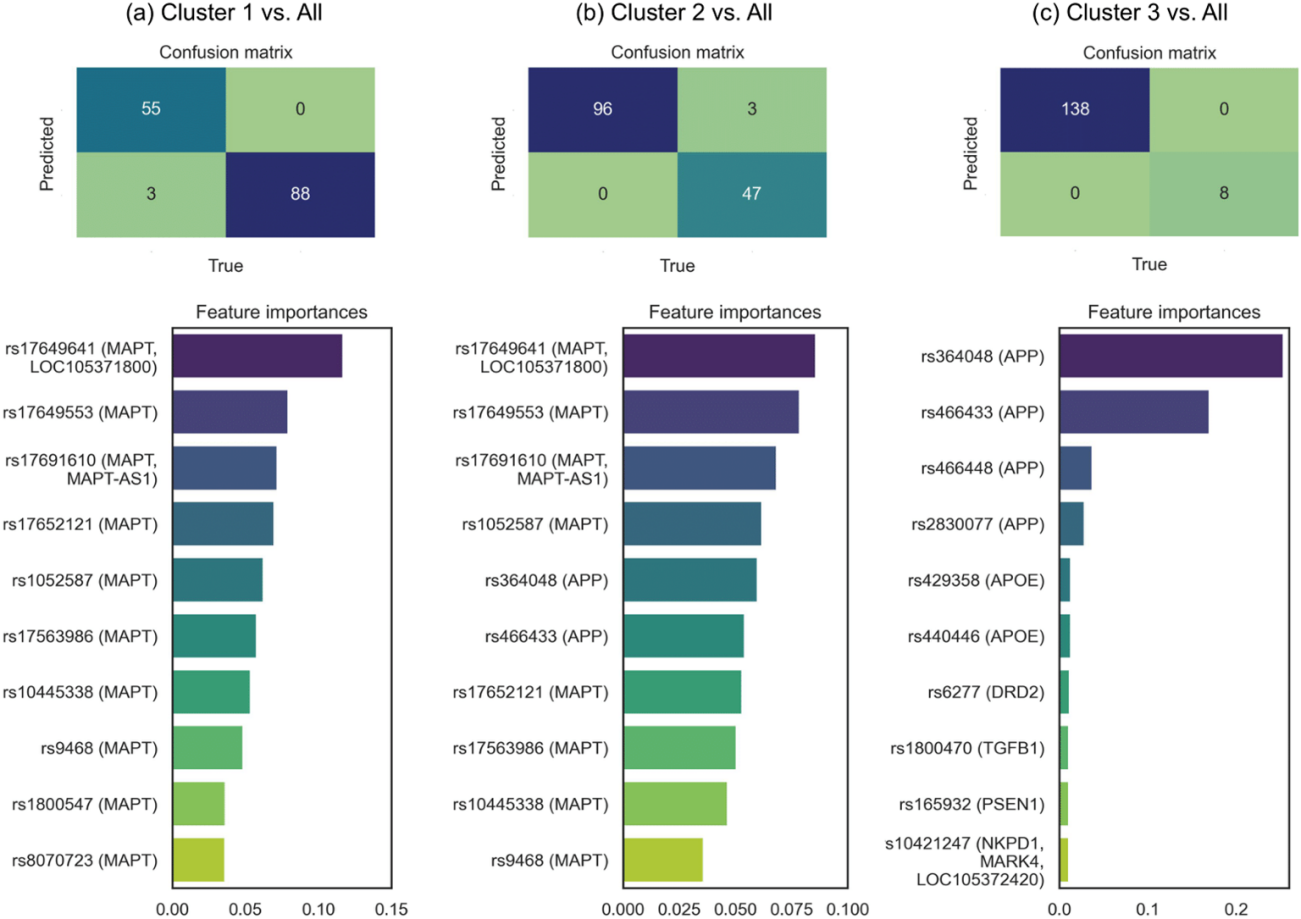
Genetic variants ranking in the classification of each cluster vs. the rest. Features importances were obtained through Random Forest, using as positive class: (**a**) Cluster 1, (**b**) Cluster 2, (**c**) Cluster 3.

Then, we analyzed which genetic variants were most important in disrupting the cluster-defining edges by means of classification models of each cluster against the other. Figure 3 shows each cluster ten most important variants for each cluster prediction and the confusion matrices obtained on the test set.

Cluster 1 results (Fig. 3a) only listed *MAPT* variants, as we showed above. Interestingly, most of these variants were intronic, with a few exceptions (such as rs17652121, which is synonymous). Furthermore, according to the VDAs selected, almost all variants in this cluster were only associated with Parkinson’s Disease (PD). Some exceptions were rs17649553, which we found only associated with AD, and rs9468, which was associated with both PD and AD but also with corticobasal degeneration and neurodegenerative disorders. On the other hand, Cluster 3 (Fig. 3c) results mainly listed variants in *APP*, although also in *APOE*, *DRD2*, *TGFB1*, *PSEN1*, or *MARK4*. Most of these variants were intronic, except for rs429358 (*APOE*), rs1800470 (*TGFB1*), and rs6277 (*DRD2*), which were missense or synonymous, respectively. In contrast to Cluster 1 list, most of these variants were associated with AD. We also found the known rs429358 variant of *APOE* associated with AD, PD, vascular dementia (VD), primary progressive aphasia (PPA), and dementia with Lewy Bodies (DLB). On the other hand, the variant found in *TGFB1* was associated only with VD. Finally, Cluster 2 variants list (Fig. 3b) resulted from combining Clusters 1 and 3 lists, mainly including variants in *MAPT* and *APP*. These results again corroborated what we observed earlier: Cluster 2 is defined through differences with Clusters 1 and 3.

### Sociodemographical, clinical, and biomarkers differences between clusters

We performed a general description of each cluster obtained by acquiring sociodemographic, clinical, biomarkers, and neurocognitive aggregated features at the baseline visit. Tables 1 and 2 summarize these variables for each cluster for Mild Cognitive Impairment (MCI) and Dementia subjects, respectively. In both tables, categorical features are shown as the number of individuals (percentage over the cluster sample), and continuous variables as mean ± standard deviation.

**Table 1:** Description of the sample of MCI individuals in the clusters obtained.

Variable	Cluster 1	Cluster 2	Cluster 3	Statistic	p-value
Number	144	268	19	NA	NA
Sex (Female)	58 (40.28%)	103 (38.43%)	58 (36.84%)	0.17	0.9175
Education years	15.92 ± 2.87	16.08 ± 2.75	16.32 ± 3.23	0.25	0.7770
Age	72.26 ± 7.60	72.57 ± 7.32	74.48 ± 6.28	0.76	0.4693
Age to *Dementia*	76.62 ± 7.67	77.05 ± 7.72	80.53 ± 4.76	0.81	0.4458
APOE E2/E2	65 (45.14%)	127 (47.39%)	12 (63.16%)	2.19	0.3350
APOE E2/E3	13 (9.03%)	17 (6.34%)	1 (5.26%)	1.12	0.5706
APOE E2/E4	6 (4.17%)	5 (1.87%)	0 (0.0%)	2.51	0.2844
APOE E3/E4	46 (31.94%)	96 (35.82%)	6 (31.58%)	0.69	0.7077
APOE E4/E4	14 (9.72%)	23 (8.58%)	0 (0.0%)	2.02	0.3639
Aβ(1–42) (pg/mL)	1021.49 ± 453.77	1000.24 ± 432.29	1010.05 ± 471.18	0.08	0.9204
tTau (pg/mL)	266.64 ± 103.30	288.73 ± 130.01	276.25 ± 78.89	1.23	0.2933
pTau (pg/mL)	25.73 ± 11.77	27.77 ± 14.55	25.57 ± 7.69	0.92	0.3990
AV45	1.23 ± 0.23	1.21 ± 0.23	1.12 ± 0.13	1.26	0.2860
FDG	1.24 ± 0.15	1.23 ± 0.13	1.24 ± 0.12	0.10	0.9069
MRI WholeBrain	0.6724 ± 0.0657	0.6846 ± 0.0729	0.6806 ± 0.0571	1.40	0.2489
MRI Ventricles	0.0260 ± 0.0153	0.0250 ± 0.0137	0.0265 ± 0.0094	0.29	0.7450
MRI MidTemp	0.0130 ± 0.0018	0.0132 ± 0.0018	0.0134 ± 0.0014	0.69	0.5000
MRI Hippocampus	0.0045 ± 0.0007	0.0045 ± 0.0007	0.0043 ± 0.0009	0.75	0.4747
MRI Fusiform	0.0116 ± 0.0016	0.0118 ± 0.0018	0.0119 ± 0.0016	0.67	0.5107
MRI Entorhinal	0.0024 ± 0.0005	0.0024 ± 0.0005	0.0021 ± 0.0005	1.69	0.1856
MMSE	27.85 ± 1.74	27.92 ± 1.59	28.58 ± 1.73	1.65	0.1930
CDR-SOB	1.50 ± 0.86	1.40 ± 0.87	1.18 ± 0.61	1.43	0.2410

*AV45* Average AV45 (Florbetapir) SUVR of frontal, anterior cingulate, precuneus, and parietal cortex relative to the cerebellum; *FDG* Average FDG-PET of angular, temporal, and posterior cingulate.

**Table 2:** Description of the sample of Dementia individuals in the clusters obtained.

Variable	Cluster 1	Cluster 2	Cluster 3	Statistic	p-value
Number	11	29	2	NA	NA
Sex (Female)	5 (45.45%)	11 (37.93%)	1 (50.0%)	0.27	0.8753
Education years	14.82 ± 1.95	15.97 ± 2.85	13.50 ± 1.50	1.34	0.2739
Age	70.91 ± 8.77	75.54 ± 8.50	85.25 ± 4.35	2.63	0.0852
APOE E2/E2	0 (0.0%)	10 (34.48%)	1 (50.0%)	5.52	0.0633
APOE E2/E3	0 (0.0%)	1 (3.45%)	0 (0.0%)	0.46	0.7948
APOE E3/E4	6 (54.55%)	15 (51.72%)	1 (50.0%)	0.03	0.9850
APOE E4/E4	5 (45.45%)	3 (10.34%)	0 (0.0%)	6.87	0.0322
Aβ(1–42) (pg/mL)	677.29 ± 399.19	714.13 ± 313.29	499.55 ± 43.55	0.38	0.6875
tTau (pg/mL)	439.24 ± 150.33	359.55 ± 101.03	262.7 ± 51.10	2.59	0.0889
pTau (pg/mL)	44.17 ± 15.49	35.39 ± 11.58	26.06 ± 5.7	2.43	0.1022
AV45	1.39 ± 0.17	1.38 ± 0.23	1.34 ± 0.063	0.04	0.9617
FDG	0.99 ± 0.12	1.02 ± 0.17	1.02 ± 0.08	0.10	0.9095
MRI WholeBrain	0.6305 ± 0.0636	0.666 ± 0.0711	0.6573 ± 0.1054	0.91	0.4109
MRI Ventricles	0.0257 ± 0.0101	0.0357 ± 0.0154	0.0195 ± 0.0000	2.28	0.1168
MRI MidTemp	0.0109 ± 0.0024	0.0118 ± 0.0019	0.0121 ± 0.0016	0.81	0.4550
MRI Hippocampus	0.0035 ± 0.0004	0.0038 ± 0.0007	0.0038 ± 0.0005	0.64	0.5348
MRI Fusiform	0.0100 ± 0.0017	0.0108 ± 0.0016	0.0109 ± 0.0002	0.95	0.3958
MRI Entorhinal	0.0017 ± 0.0003	0.0020 ± 0.0005	0.0023 ± 0.0002	2.18	0.1277
MMSE	22.45 ± 1.72	22.90 ± 2.02	22.50 ± 0.50	0.22	0.8047
CDR-SOB	5.45 ± 1.78	4.07 ± 1.42	4.25 ± 0.75	3.18	0.0523

*AV45* Average AV45 (Florbetapir) SUVR of frontal, anterior cingulate, precuneus, and parietal cortex relative to the cerebellum, *FDG* Average FDG-PET of angular, temporal, and posterior cingulate.

None of the consulted variables significantly differed among MCI subjects (Table 1), although we observed some statistically significant trends between Dementia subjects (Table 2). Dementia subjects showed more significant differences, although these were much smaller groups than the MCIs, especially in the case of Cluster 3.

Considering socio-demographic characteristics, the only two Dementia subjects in Cluster 3 were almost significantly ten years older than those in the other two clusters (85.25 ± 4.35, p-value 0.0852, Table 2). This trend could also be observed in the MCI individuals in Cluster 3, which, although revealing no significant differences, had the highest mean age at which they received the diagnosis of Dementia (80.53 ± 4.7605, p-value 0.4458, Table 1). We also observed differences close to the significance threshold regarding *APOE* genotypes, with most E2/E2 subjects in Cluster 2 (p-value 0.0633, Table 2). None of the Dementia subjects in Cluster 3 showed the highest risk genotype for APOE (E4/E4), a difference that obtained a significant p-value (0.0322, Table 2). This trend could also be observed in Cluster 3 MCI individuals, of which none showed the E4/E4 genotype (Table 1). Regarding CSF biomarker values, Cluster 1 was the one that showed a trend towards higher tau values. Total tau (tTau) mean value in Cluster 1 was the highest (439.24 ± 150.33) and obtained near significant differences (p-value 0. 0889, Table 2), as did those of pTau (44.17 ± 15.49, p-value 0.1022, Table 2). Finally, we found that Dementia subjects in Cluster 1 showed the highest CDR-SOB mean value (5.45 ± 1.78, p-value 0.0523, Table 2).

### Baseline and longitudinal hypometabolism profiles

We then explored the hypometabolism profile of each genetic cluster using FDG-PET neuroimaging. Using Linear Mixed Models (LMMs), at baseline and longitudinally (considering the follow-up time). As in the previous Section, we performed this analysis selecting MCI and Dementia subjects (Supplementary Table 2).

**Figure 4:**
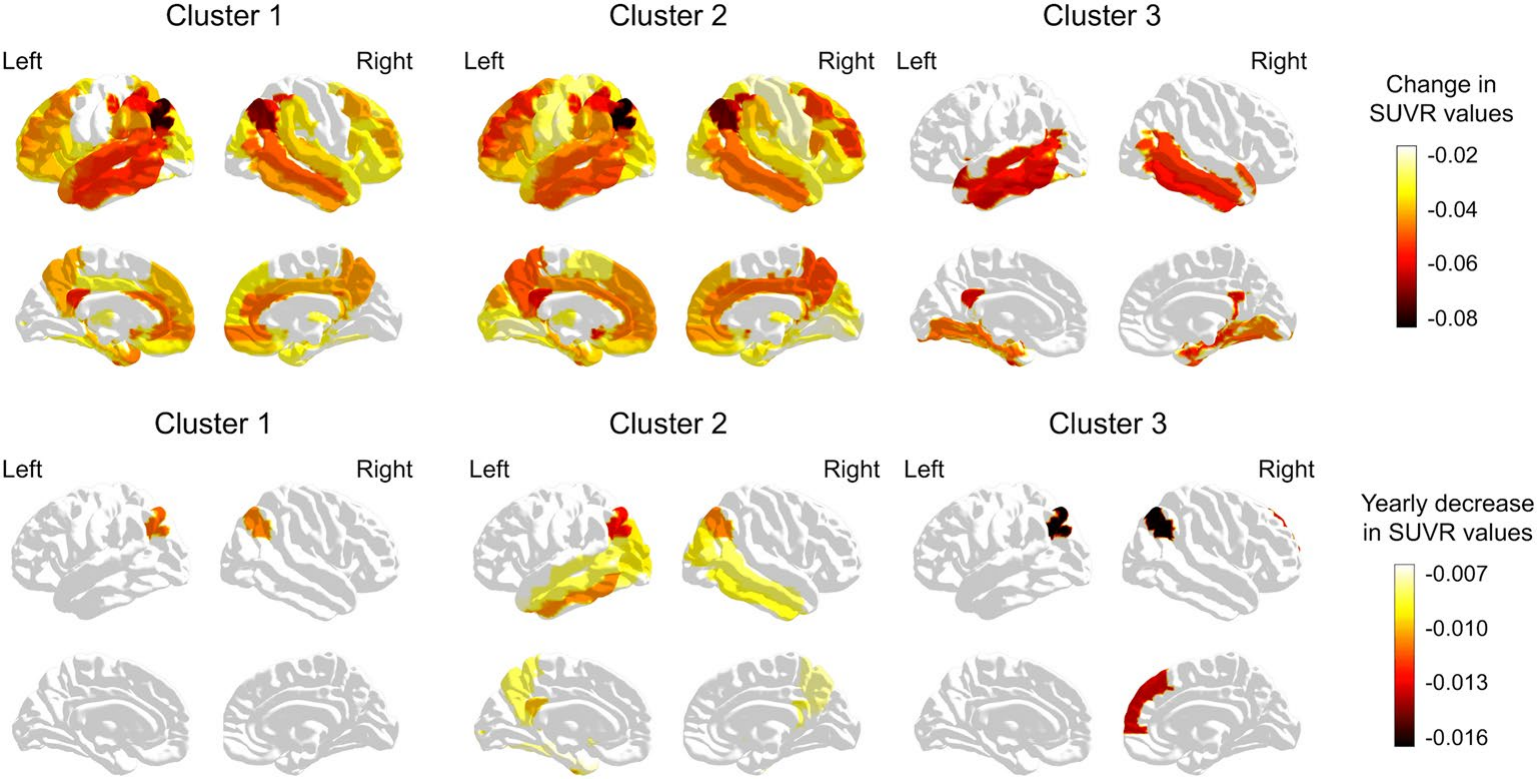
Average metabolism coefficients against controls in each cluster. (**a**) Baseline, and (**b**) longitudinal analysis. ROIs correspond to the AAL atlas^26^. The shown regions obtained a p-value (FDR corrected) < 0.05.

Figure 4 shows how much, on average, Standardized Uptake Value Ratios (SUVRs) differ from controls for each significant Region Of Interest (ROI) at baseline (Fig. 4a) and longitudinally (Fig. 4b). Supplementary Tables 3 and 4 also list these significant ROIs, including the LLMs models coefficients, and statistics. To ease the analysis, we employed classification presented in Ref.^25^ to group the significant regions. Moreover, in the following, we will describe each ROI according to the abbreviations defined by Ref.^26^, also specifying for left (L), right (R), or both (L&R) hemispheres.

Overall, Fig. 4 shows a clear difference in the number of significantly affected regions between Cluster 3 and the other clusters, which is most likely due to its low sample size. At baseline (Fig. 4a), we found fifteen regions affected in all clusters, all of them temporal (MTG L&R, ITG L&R, TPOsup L&R, HIP L&R, PHG L&R, FFG L&R, and AMYG L), except a parietal region (PCC L&R). Clusters 1 and 2, much larger than Cluster 3, had more significantly affected regions, of which fifty-one were in common: more than half frontal, prefrontal, and other frontal regions. Cluster 2, the one with the largest sample size, was the only one with unique significantly affected regions, which included eight occipital (CUN L&R, IOG inf L&R, SOG L&R, and CAL L&R), five parietal (SPG R, PreCG LR, PoCG LR) and two frontal (SMA L, ROL R).

The most affected regions (coefficient ≤ − 0.05) in all clusters were the left posterior cingulate gyrus (parietal and limbic system) and the left middle and inferior temporal gyrus (temporal regions). Cluster 1 showed more affected parietal and temporal regions, and one pre-frontal (CAU L). Cluster 2 was similar to Cluster 1, although it included more affected prefrontal regions (such as CAU L&R or MFG L&R). Lastly, Cluster 3 also included a parietal region (PCC L&R) as highly affected. The rest of most affected regions in Cluster 3 were parietal, much also being part of the limbic system (such as the hippocampus, parahippocampal region, or the amygdala).

The longitudinal analysis allowed us to analyze whether the new groups of subjects determined by genetic characteristics presented different trajectories in the evolution of their hypometabolic profiles. According to this analysis (Fig. 4b), Cluster 2 showed the most aggressive neuroimaging course, with more regions displaying hypometabolism over time, followed by Clusters 3 and 1. The angular (parietal) regions changed significantly over time in all clusters. Cluster 1 only included these angular regions as significant. Cluster 2 also included many temporal (ITG L&R, MTG L&R, TPOmid L, PHG L, and FFG L) and parietal (PCC L&R, and PCUN L&R) regions, and some occipital (MOG L&R and IOG L) and prefrontal (CAU L&R) ones. Finally, Cluster 3 also included a prefrontal region (SFGmedial R).

Therefore, we observed that Clusters 1 and 2 had more regions significantly affected than controls at baseline than Cluster 3. Cluster 1 mainly had parietal and temporal regions affected, Cluster 2 was similar to Cluster 1 but included frontal regions, and finally, Cluster 3 had a more contained but more affected hypometabolism, mainly in temporal regions belonging to the limbic system. At longitudinal, all clusters had hypometabolism in the angular parietal region. In addition, Cluster 2 also included occipital, temporal, and parietal regions, and Cluster 3 also prefrontal one.

### Graph-based cognitive profiles

Finally, we analyzed the neurocognitive data from a recent novel perspective: network neuropsychology^27^. In these approaches, graph theory and network modeling are applied to neuropsychological test data to deliver new knowledge on these diseases’ profiling and cognitive functioning^27^. With this strategy, our conception was to provide more detailed cognitive profiling of each cluster obtained and provide a comprehensive view of the genetic profiles described.

For each cluster, we built a neurocognitive network based on the Alzheimer’s Disease Assessment Scale–Cognitive Subscale (ADAS-Cog) neurocognitive test, at baseline. We chose the ADAS-Cog test ahead of others, such as Mini-Mental State Examination (MMSE) or Montreal Cognitive Assessment (MoCA), because it is more complete and is not used as much for pre-screening tasks as the mentioned ones. In addition, ADAS-Cog mainly focuses on the neurocognitive domain of memory, especially relevant regarding the considered cohort in which potentially most individuals will convert to AD, i.e., mainly with memory impairment. However, we speculate that these subjects do not reach this symptomatology through the same pathways, based on their genetic-level perturbations.

**Figure 5:**
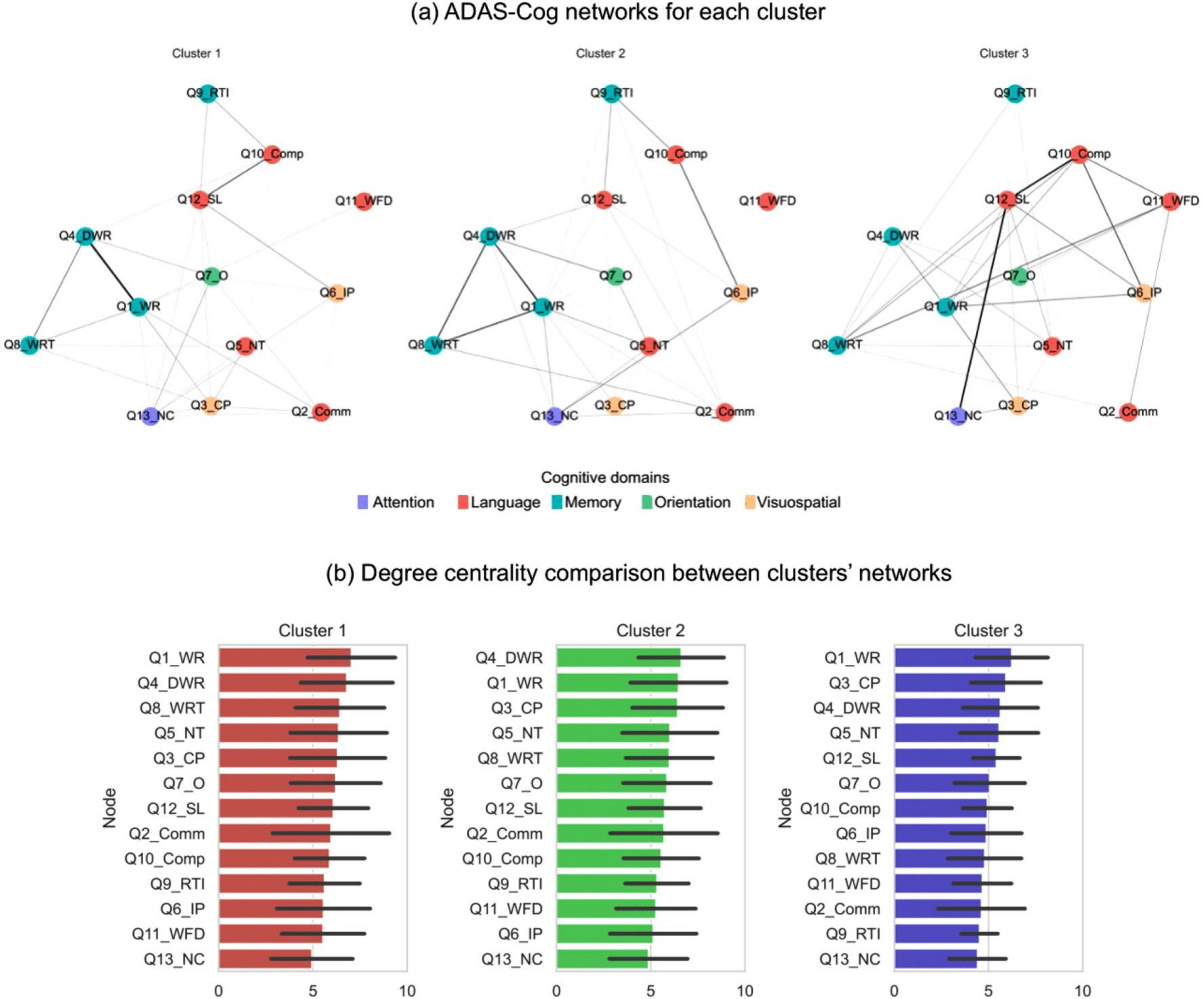
ADAS-Cog neurocognitive networks results for each cluster. (**a**) Neurocognitive networks built for each cluster; nodes are colored according to the cognitive domain that the item or test they represent. Edges are colored and weighted according to the edge weight. (**b**) Degree centrality of nodes in clusters’ cognitive networks. Edges weights and degree centrality values were obtained through bootstrap calculation (n = 10, 250 repeats).

As in the previous sections, we selected those subjects with MCI or Dementia diagnosis at baseline. Concerning the availability of the ADAS-Cog data for each subject and their baseline diagnosis, the sample size included in this analysis was 104, 212, and 15 for Clusters 1, 2, and 3, respectively. The resulting networks represent the weighted interactions (edges) between each item (nodes) of the neurocognitive test Fig. 5 shows each cluster cognitive network (Fig. 5a) and the degree centrality distributions of the test items (Fig. 5b). Supplementary Table 5 shows the global graph metrics computed for each cluster’s cognitive network and their statistical comparison.

Generally, all clusters’ networks showed different topologies (Fig. 5a), which we corroborated by computing global graph measures (Supplementary Table 5). All the measures consulted (diameter, density, average degree, and average clustering coefficient) significantly differed between clusters (p-value < 0.05, ANOVA + Tukey’s HSD), especially between Cluster 3 and the other two clusters. Cluster 3 cognitive network was the most different. Cluster 1 network tended to be less extensive (smaller diameter), denser (higher density and average degree), and more likely to form communities (higher average clustering coefficient). The most weighted connections in the three networks were generally between items belonging to the same cognitive domain (showing the same node color in Fig. 5a), such as items “Word Recall” (Q4_DWR), “Word Recall” (Q1_WR), and “Word Recognition Task” (Q8_WRT), which correspond to the memory cognitive domain (cyan).

Lastly, nodes’ degree centrality measures (Fig. 5b) of each cognitive network did not reveal many similarities between the clusters according to their cognitive profile. In all clusters, memory items “Word Recall” (Q1_WR) and “Delayed Word Recall” (Q4_DWR) were the most central (higher degree). In addition, other items were central (with slightly different positions between clusters) related to language and visuospatial cognitive domains such as “Naming Task” (Q5_NT) or “Constructional Praxis” (Q3_CP), respectively.

## Discussion

In this work, we have implemented and evaluated a methodology for stratifying patients based on their genetic characteristics using network biology and graph analysis strategies. Employing ADNI’s genetic cohort, we developed a methodology to compute perturbation scores of a brain-specific PPI for each individual. Employing these scores as input, we clustered the cohort subjects based on their similarity using a spectral clustering model. Finally, we profiled the discovered clusters based on their genetic characteristics and the pathophysiological and symptomatological features they present, with the primary objective of defining distinct subgroups of AD patients.

Based on genetic alterations mapped into a brain-specific PPI network, we discovered three clusters, named Cluster 1, 2, and 3, respectively. These clusters were defined primarily by edge perturbations present in two genes with a high centrality degree (i.e., number of interactions) within the original PPI: *MAPT* (microtubule-associated protein tau) and *APP* (amyloid precursor protein). Interestingly, these two genes code for the two proteins altered within AD pathology: tau and amyloid proteins. Cluster 1 had a high degree of affectation regarding *MAPT* interactions, Cluster 3 in *APP*, and Cluster 2 in neither of these mentioned genes.

The involvement of *MAPT* interactions in Cluster 1 was mainly generated by genetic variants such as rs17649641 or rs17649553, previously associated with PD and its age of onset in familial studies^28–30^. The relationship of rs17649553 with AD has also been studied in the Chinese population, although no significant results were obtained^31^. Additionally, some of the *MAPT* variants found in this cluster tag different haplotypes of this gene, such as the H1, associated with a higher tau expression as well as with the AD, PD, frontotemporal dementia (FTD), and amyotrophic lateral sclerosis (ALS)^32^. We also found other relevant variants, such as rs9468, associated with neurodegenerative disorders, AD, and corticobasal degeneration^33–35^. Another interesting variant was rs8070723, associated with progressive supranuclear palsy (PSP), a tauopathy that causes movement disorders^36^. This variant also had a modest significant association with behavioral FTD and nonfluent PPA^37^. Notably, according to the most altered gene, *MAPT*, Cluster 1 showed higher CSF tTau and pTau values in subjects with a dementia diagnosis, a result previously observed in other similar neurodegenerative diseases^32^, as mentioned above. Cluster 1 showed the lowest scores for the interactions with this *APOE* (Supplementary Table 1). This result was also reflected in the percentage of risk APOE genotypes such as E2/E4 and E4/E4, which were the highest in Cluster 1 Dementia and MCI subjects, respectively (Tables 1 and 2). Thus, in addition to tau gene alterations, this cluster showed a higher risk of developing dementia and amyloid formation^38^. Finally, this cluster showed more symptomatically affected Dementia subjects, i.e. showing the highest CDR-SOB value (Table 2).

Cluster 2 included the largest sample from the original cohort (456, 63%). It was mainly defined through the differences between the other clusters, corroborated by observing the most important variants for the characterization of this cluster (Fig. 3), a mixture of the most important ones in Clusters 1 and 3. We only found one significantly altered interaction in this cluster, MC1R-MAPK7. In these genes, we found several variants, such as the rs2228479 missense variant (*MC1R*), associated with an increased risk of developing late-onset AD, especially in subjects whose genetic risk could not be explained by the *APOE* genotype^39^. This result is especially intriguing for Cluster 2 since, even with the largest sample size, we found no significant differences against the other clusters regarding risk E4 *APOE* genotypes in either the MCI or Dementia individuals (Tables 1 and 2).

Above all, Cluster 3 was mainly defined by alterations in *APP*. Genetic variants in this gene are usually associated with familial and dominant AD^38^, although we did not find these in this cluster. Instead, we found several rare *APP* variants whose apparition has been previously described as associated with the risk of developing sporadic AD^38^. The fact that they are considered "rare" variants was probably the reason for this cluster’s sample size, which had the fewest subjects from the original sample (38, 5% of the total). Cluster 3 size was one of the main limitations of its description since it prevented adequate profiling of the patients in this group. Among the *APP* variants important in defining this cluster (Fig. 3), rs364048 was associated with a risk of developing sporadic ALS^40^. Moreover, rs466433 and rs364048 have been described as protective variants in an AD Han Chinese population^41^, although this result could not be replicated in a Caucasian population GWAS^42^. rs466448 was previously associated with early and late-onset AD^43^ and beta-amyloid CSF levels in PD^44^. Finally, the rs2830077 variant was associated with verbal and total IQ in children and AD cognitive impairment in adults^45^. In addition to *APP* variants, we also found the well-known rs429358 *APOE* variant necessary for this cluster’s definition. This variant is the one that defines the ε4 allele, and it is associated with an increased risk of developing AD^38^. However, Cluster 3 showed the opposite behavior to Cluster 1 regarding this gene, where we only found a single E4 allele carrier (Tables 1 and 2), meaning a low risk for developing AD dementia. This result was also reflected in Cluster 3 *APOE* interaction scores, which showed the highest mean (Supplementary Table 1) except for the APOE-APP interaction. Therefore, although Cluster 3 involved a series of perturbations on a high-degree gene such as *APP*, it appeared less associated with the risk of developing AD. Likely related to this lower risk, Cluster 3 showed a trend towards a higher mean age at conversion to Dementia diagnosis (Tables 1 and 2), although not significant. Lastly, we found other variants important for this cluster's characterization in amyloid processing genes, such as presenilin 1 (*PSEN1*).

Many of the variants described here have not previously been described as associated with AD, meaning that they have not been demonstrated as pathogenic in isolation, regarding previous linkage or GWAS studies. However, given that the protein their gene code for most likely has a role in the pathophysiology of the disease (especially in the case of *MAPT* and *APP*), the results here presented also offer this methodology a potential new way to find variants and genes related to the pathophysiology as mentioned earlier, which cannot be detected only employing more traditional methodologies. Furthermore, in many cases, many of the variants reported were found to be associated with or related to neurodegenerative diseases different from AD, confirming that these groups have in common a neurodegeneration profile. Additionally, the observed genetic profiles suggested that *MAPT* (tau) has a more relevant role at a genetic level than *APP* (amyloid). Therefore, this suggests that, in the absence of a known autosomal-dominant causal mutation in *PSEN1*, *PSEN2*, or *APP*, genetics would have a more relevant role at the level of the amyloid hypothesis cascade related to tau and neurodegeneration, and perhaps not so much in the early events related to amyloid deposition.

Regarding the hypometabolism profile (Fig. 4), all clusters showed several significantly affected regions in common, both basally and longitudinally, mainly temporal and parietal. Among the most affected regions common to all clusters were the cingulate gyrus region and some parietal lobe regions. This parietal-temporal pattern is typical of AD^12,46,47^, corroborating this diagnosis in all clusters. Clusters 1 and 2 were more similar and showed more significantly affected regions than Cluster 3 when compared to controls. Cluster 1 had a pattern of involvement considered classic, with hypometabolism involvement concentrated in the parietal-temporal regions, with more affectation in the parietal ones. In addition, Cluster 2 had particularly affected prefrontal regions, a profile similar to the so-called "limbic-predominant" in a FDG-PET image clustering work^12^. This “limbic-predominant” group was associated with older age compared to the more typical hypometabolism pattern, as is the case among individuals with dementia in Clusters 1 and 2 (Table 2). In addition, this prefrontal hypometabolism has previously been associated with amyloid accumulation in remote regions^48^. Cluster 3 also presented a much more contained pattern of hypometabolism, especially in temporal and limbic system regions. Despite the differences described, the patterns observed were probably very dependent on the sample size obtained for each group and the time of assessment. In this sense, it would be interesting to cross-check the results obtained with works such as those presented by^12,45,49^, in which a typical AD profile (in our case probably related to Cluster 1) and a predominantly limbic profile (probably more associated with our Clusters 2 and 3) are generally observed. Finally, although all groups showed a pattern of parietal-temporal involvement with posterior cingulate involvement characteristic of AD, they all appeared to have different neuroimaging evolutions, which could suggest the described genetic profiles as the ground for different disease trajectories. Moreover, in the future, it would be valuable and highly informative to inspect other methodologies for performing this neuroimaging profiling, such as performing a voxel-wise analysis of the whole brain metabolism.

Finally, the groups presented a similar neurocognitive profile among patients (Fig. 5). Given that the hypometabolic profiles were similar, except for a few regions, the expected result was that the neurocognitive networks constructed would not differ too much. In this case, they all presented memory items as the most central and characteristic ones, a typical pattern observed in AD cognitive networks^50^. We observed minimal differences, similar to what was observed in the neuroimaging profile, with visuospatial domain items relatively more central in Clusters 2 and 3. The fact that we did not observe especially relevant differences in cognitive or neuroimaging profiles at baseline seems logical because the same disease stage (MCI) was selected as a starting point and cognitive tests available are focused on the characteristic cognitive functions impaired in AD.

The obtained results indicated three genetic subtypes of the disease, all showing typical characteristics associated with AD such as tau and beta-amyloid accumulation, parieto-temporal hypometabolism profiles and memory-centered cognitive profiles. The found homogeneity when phenotyping these groups may come from the initial homogeneity present in the ADNI cohort, focused on the study of AD, and in which it is difficult to find other pathologies or subtypes of non-classical AD. On the other hand, this homogeneity could also indicate that, although there is a common disease development, there are diverse genetic pathways by which the classical AD phenotype can be reached. This fact has been suggested by other works, in which it is postulated that genetic variants affecting at different levels of common pathways (such as *APOE*) may lead to aggregation and co-occurrence of tau and beta-amyloid^51^. Despite finding many similarities between the presented clusters, each of them presented some defining features. Cluster 1 mainly presented alterations in *MAPT* and a higher percentage of people with *APOE* e4 allele (therefore, with a higher risk of developing dementia due to AD), as well as higher tau levels at the dementia stage. Cluster 2 was an intermediate cluster between 1 and 3, also with respect to *APOE* allele frequency. This "intermediate" genetic pattern was also reflected in FDG-PET of Cluster 2 which showed a more heterogeneous hypometabolism with more regions affected than the other two clusters. Finally, Cluster 3 appears to be a lower risk group for the development of dementia due to AD, defined mainly by rare (perhaps protective) variants in *APP* and almost no *APOE* ε4 individuals. This group was the one with the highest age at the development of dementia, with a much more contained hypometabolism.

There are some limitations in this work development. First, the low sample size of the *APP*-related cluster prevented us from adequately profiling its physiopathological and neurocognitive characteristics. In the future, this subgroup should be evaluated with a larger sample size, for example, using a larger genetic cohort than the one used in this case. However, note that if the mutations found in this subgroup are rare, obtaining an adequate volume of subjects to obtain information on this genetic group will be difficult. Second, although the sample size of the cohort used in this work is adequate (> 700 samples), it would be necessary to evaluate the clustering results obtained in a so-called "validation cohort". In this regard, novel cohorts, including genetics, neuroimaging, and cognitive follow-up, are currently under development and could be available shortly. Third, although we included a broad set of variants, both associated with AD and many other related neurodegenerative diseases, knowledge-based strategies that rely on curated data such our work are prone to include biases of multiple types (selection, population, publication, etc.). In the future, it would be highly valuable to inspect these biases, e.g. by introducing more non-previously associated variants, or evaluating the obtained clusters in different cohorts coming from different populations. Furthermore, regarding the hypometabolism analysis, it is important to note that we performed the intensity normalization using the cerebellum as the reference region, since it is still one of the most used regions in AD and is suitable in early stages of the disease^52^. However, in the future, it would be interesting to explore these profiles using other more advanced normalization methods^53,54^. Finally, and very importantly, despite developing this work with a cohort dedicated to the study and research of AD (ADNI), the stratification analysis developed could be influenced by different pathologic substrates or comorbidities present. In this sense, we found in the considered cohort a very low percentage of MCI or dementia due to non-AD (about 4% according to the ADNI diagnostic information). It would be necessary in the future to more deeply evaluate the possibility of this phenomenon using different types of biomarkers and neurocognitive tests.

Finally, in addition to the clinical consequences within AD, this work proposes a novel methodology combining machine learning and systems biology, approaching the study of neurodegenerative mechanisms holistically and not so focused on specific proteinopathies. Thus, the edge scores employed were computed by mapping the accumulation of mutations on each of the edges of a biological network (in this case, a PPI network). These edge scores did not only give importance to the mutations accumulated in each considered gene, but also to the interactions between them. By doing this, we made sure of quantifying the effect of these perturbations (genetic variants) on the underlying disease’s biological processes themselves, and not only on the actors (genes/proteins) of these processes. In this sense, we have carried out a straightforward strategy, including a mutation accumulation score on a common biological network such a PPI, although in the proposed methodology it could be easy to include more data such as pathogenicity scores or other types of biological interactions.

In conclusion, our work suggests the presence of distinct genetic clusters, defined mainly by alterations in gene interactions of the characteristic proteins of AD, *MAPT*, and *APP*, as well as the known risk alleles of the *APOE* gene. Finally, the fact that we found both a tau- and an amyloid-predominant group could suggest different onset mechanisms or a more relevant role of one pathway or the other. Likewise, the existence of clusters from the genetic point of view, together with the fact that all of them reach the same disease by different mechanisms, suggests the possibility that these groups have different topographic and prognostic trajectories, given that each gene can put greater vulnerability of specific regions.

The patient stratification analysis performed in this work allowed us to describe different genetic architectures for the onset of AD disease, a key starting point for the development of personalized therapeutic targets for Alzheimer's disease (AD). Our findings reveal little variation in genetic profiles related to AD symptoms and pathology, highlighting the fact that distinct biological mechanisms could lead to similar symptomatological and pathological changes, even the same disease diagnosis. Consequently, treatments should target the unique genetic architecture of each subgroup rather than adopting a universal drug development strategy. Moreover, our study pinpoints the main genes (MAPT, APOE, and APP) where these genetic differences appear, proposing them as potential targets for personalized drug development for each AD-subgroup.

## Materials and methods

### Genetic data preprocessing and filtering

We selected Variant-Disease Associations (VDAs) from DisGeNET^55^, associated with the following diseases (CUI codes in parenthesis): “Alzheimer’s Disease” (C0002395), “Neurodegenerative Disorder” (C0524851), “CNS degeneration” (C0262424), “Degenerative disease of the central nervous system” (C0270715), “Frontotemporal dementia” (C0338451), “Parkinson’s Disease” (C0030567), “Corticobasal degeneration” (C0393570), “Vascular dementia” (C0011269), “Primary progressive aphasia” (C0282513), and “Lewy body dementia” (C0752347). The total number of unique VDAs was 2924.

Next, we queried for these variants in the Whole-Genome Sequencing (WGS) cohort from the Alzheimer’s Disease Neuroimaging Initiative (ADNI) (adni.loni.usc.edu). The ADNI database was launched in 2003 as a public–private partnership, led by Principal Investigator Michael W. Weiner, MD, has as its primary goal to test the combination of several neuroimaging techniques, biological markers, and clinical and neuropsychological evaluations to assess the progression of Mild Cognitive Impairment (MCI) and early AD. ADNI’s WGS cohort consists of 808 subjects. Querying for the mentioned variants of interest in this cohort, resulted in a total of 2475 variants located in a total of 1071 genes. In addition, to avoid clustering phenomena associated with stratification by genetic population, we selected only subjects in the cohort who had a Caucasian ancestry (“Not Hispanic/Latino” and “White” values regarding “PTEHTCAT” and “PTRACCAT” variables in “ADNIMERGE” key table), resulting in 728 subjects.

### Patient-specific edge scores creation

We then organized genes from selected variants into a subnetwork of PPIs querying the brain tissue-specific PPT-Ohmnet network from the Stanford Biomedical Network Dataset Collection^22^. We reduced the original number of genes, filtering out genes where we did not find exonic variants. The resulting PPI subnetwork, used as a starting point for further processing and analysis, consisted of 66 nodes and 102 edges. Next, we generated a weighted graph for each patient based on the aforementioned PPI subnetwork, computing PPI’s edge scores based on the obtained genetic variants data. First, for each patient, for each of the edges present in the PPI subnetwork, we computed the percentage of variants present in the two interacting genes w.r.t. the total number of mutations found in the initial cohort. This value represented the degree to which an edge is affected as a function of the accumulation of mutations w.r.t. the entire cohort. For example, a subject showed 2 and 4 mutations in interacting genes A and B, of which a maximum of 4 and 6 variants were found in the whole cohort. Thus, resulting in a score of 0.6 ([2 + 4]/[4 + 6]). Secondly, we assigned a conservation score to each edge, defined as the weight of the unaffected edge (1) minus the previously computed mutation value. In the previous example, the final edge score will be 0.4 (1 − 0.6). This final edge score functions as a representation of the conservation degree of the original interaction between two genes, i.e., the degree to which that edge is maintained concerning the original network. Following this formula, we obtained a dataset of weighted graphs for the considered genetic cohort. Therefore, we ended up with a matrix of subjects (rows) × edges (columns), where values in the range of zero to one represented the conservation of the original edge. A value of zero corresponded to a fully affected edge, in which, for that patient, all mutations found in the two interacting genes were found, and a value of one represented an unaltered interaction in which no mutations were found in that patient. We ended up with a total of **728** subjects whose genetic characteristics were distributed across **102** variables, corresponding to the weights on the 102 edges of the original PPI brain-specific subnetwork considered.

### Clustering method and evaluation

Using as input the matrix of edge scores presented above, we obtained subgroups of patients employing a clustering strategy based on Similarity Network Fusion (SNF), a method first introduced by Ref.^56^. Although SNF was initially conceived to combine several types of information, we only performed this clustering using the edge scores dataset. SNF builds a distance matrix (using a given metric) between samples, which is converted to an affinity matrix based on the similarity of a subject to its *K*-neighbors. *K* is one of the hyperparameters of this algorithm, and it determines the number of close neighbors to be considered when constructing the affinity matrix. Another hyperparameter called *μ* is a factor that weights the affinity matrix. The affinity matrix between subjects can be thought of as a similarity graph, where similar samples will show a higher weighted connection. Considering this network of subjects, the next step is to apply a spectral clustering strategy to find groups of nodes (subjects) that could be considered similar based on their genetic information.

We employed a Python implementation of SNF, named SNFPy^57,58^, based on the original code developed by Ref.^56^. We employed the squared Euclidean distance to build the first distance matrix. According to SNFPy’s documentation, *K* has to be around the total number of samples divided by 10 and μ between 0.2 and 0.8. To select the solution with the most appropriate and most interesting number of clusters, we set *K* to 70 (approximately the total number of samples divided by 10) and performed a grid search of the μ hyperparameter (from 0.2 to 0.8) for a range of clusters (from 2 to 10). To assess the best clustering solution, we computed the corresponding Silhouette Index (SI) score. It should be noted that this SI score is implemented with some modifications w.r.t. the original one^57^ in order to take into account a similarity matrix rather than distances. For more information on the modified implementation of this clustering metric, visit SNFPy’s documentation^57^.

### Genetic description of the clusters: classification models using cluster labels

We performed several steps for listing the most important genetic variants for defining each cluster. First, we selected those interactions (edges) that presented a significantly different (p-value < 0.05) score between the obtained clusters, employing ANOVA followed by a Tukey’s HSD test. Secondly, we extracted the genetic variants present in the genes that were found to be interactors of significantly different interactions to assess their predictive ability and significance. Using these selected variants as input features, we implemented a Random Forest classification model for each cluster *vs.* all others, i.e., three classification models. For each classification model, we divided the original sample into stratified training (80%) and test (20%) sets, i.e., taking into account the original distribution of the class (cluster). To avoid classification problems regarding clusters’ sample distributions, we employed a Random Forest implementation that accounts for class imbalance^59^. Lastly, we extracted the feature importance values from the Random Forest models and reported the 10 most important genetic variants to define each cluster.

### Sociodemographical and biomarkers description

For profiling the genetic clusters, we first performed a complete description of each of them regarding several sociodemographical and biomarkers measurements available for the considered cohort. For this, we obtained features from the “ADNIMERGE” key table from ADNI, representing sociodemographic data (gender, age at baseline, education years, and ethnicity), APOE genotypes, aggregated neuroimaging measures (amyloid and FDG-PET), CSF biomarkers measurements (Aβ 1 − 42, tTau, and pTau), and total neurocognitive tests scores (MMSE and CDR-SOB). Importantly, we restricted the analysis only to MCI and Dementia subjects (diagnosis at baseline) to assess whether each cluster’s genetic characteristics were important in defining the disease spectrum at the onset of the clinical disease. For the comparison between clusters we employed a Chi-square test for the categorical features, and ANOVA followed by a Tukey’s HSD test for the continuous variables. We considered significant differences for a p-value < 0.05.

### Hypometabolism-specific neuroimaging profiling

We performed a neuroimaging analysis to discover differences in the hypometabolism patterns of each cluster defined through genetic information. For each patient, we selected the FDG-PET images obtained closest to the baseline visit and in the following years. We downloaded preprocessed images from ADNI (co-registered and averaged). Next, we processed the images using Statistical Parametric Mapping (SPM) software^60^ implemented in Matlab 2020b (MathWorks Inc.) through the Python interface “nipype”. We realigned and normalized to the Montreal Neurological Institute space using the FDG-PET template presented in Ref.^61^ to voxels of size [2, 2, 2] using a 7th Degree B-Spline interpolation. Afterward, we applied spatial smoothing with a Gaussian kernel of 6 mm of Full Width at Half Maximum (FWHM). Subsequently, we aggregated the brain metabolism data into 116 regions of interest (ROIs) based on the AAL atlas^26^. Finally, we calculated SUVRs using the whole cerebellum as the reference region. We conducted LMMs to evaluate if the cluster subjects presented different ROIs metabolism against control subjects at (i) baseline and (ii) during the time (longitudinal analysis). As a dependent variable, we considered the SUVR values of each ROIs, and sex and age as covariates. In addition, for the longitudinal analysis, we evaluated the interaction effect between the cluster membership variable (coded as a dummy variable) and the follow-up time. This interaction analysis permitted us to analyze whether clusters present different brain metabolism trajectories. Therefore, we selected ROIs that showed significantly different (FDR corrected p-value < 0.05) hypometabolism values (i) between clusters and (ii) between clusters taking into account follow-up time.

### Neurocognitive profiling

When available, we obtained each subject’s data from the baseline visits from ADAS-Cog neurocognitive test^62^. ADAS-Cog comprises 13 items that assess functions pertaining to five cognitive domains (memory, language, visuospatial, attention, and orientation). Supplementary Table 6 shows the items (nodes) of which ADAS-Cog consists, as well as the main cognitive domain to which each of these items is associated and their range score. We built a partial correlations matrix for each genetic cluster, using each subject’s subscores as input. The resulting correlation values represented the weighted interactions (edges) between each item (nodes). We ended up with a neurocognitive network for each genetic cluster. Because the sample size in the case of some clusters was small, we obtained these correlations by applying a bootstrap technique to avoid biases related to this. For this, we selected a specific number of subjects (n = 10) and obtained the correlation matrix for that subgroup. We repeated this process 250 times for each cluster. Therefore, the weights of the interactions between items of the presented neurocognitive networks represented the mean of this bootstrap analysis. In addition, to characterize each of these networks, we obtained the mean values of diameter, density, and average clustering coefficient for each of the bootstrap repetitions for each cluster, compared through ANOVA and Tukey’s HSD tests. Thus, we could compare whether the topology of these neurocognitive networks differed between clusters. Finally, we also obtained the average measures of the degree centrality of the nodes (items) to describe which cognitive functions or domains were most important in each of the clusters and whether there were differences on this basis.

### Supplementary Information

Supplementary Information.

Supplementary Table 3.

Supplementary Table 4.

## Data Availability

Data used in the preparation of this article were obtained from the Alzheimer’s Disease Neuroimaging Initiative (ADNI) database, which is available under a formal request (adni.loni.usc.edu). The code developed for this work can be found at the following repository: https://github.com/laurahdezlorenzo/AD_genetics_stratification.
