# Author Correction: Genetic-based patient stratification in Alzheimer’s disease

**DOI:** 10.1038/s41598-024-62454-9

**Published:** 2024-05-21

**Authors:** Laura Hernández-Lorenzo, Fernando García-Gutiérrez, Ana Solbas-Casajús, Silvia Corrochano, Jordi A. Matías-Guiu, Jose L. Ayala

**Affiliations:** 1 Department of Computer Architecture and Automation, Computer Science Faculty, Complutense University of Madrid, 28040 Madrid, Spain; 2 Department of Neurology, San Carlos Research Institute (IdSSC), Hospital Clínico San Carlos, 28040 Madrid, Spain; 3 Instituto de Tecnología del Conocimiento, Universidad Complutense de Madrid, 28040 Madrid, Spain

Correction to: *Scientific Reports* 10.1038/s41598-024-60707-1, published online 30 April 2024

The original version of this Article omitted an affiliation for Jose L. Ayala. The correct affiliations are listed below.

Department of Computer Architecture and Automation, Computer Science Faculty, Complutense University of Madrid, 28040, Madrid, Spain

Instituto de Tecnología del Conocimiento, Universidad Complutense de Madrid, 28040, Madrid, Spain

The original Article has been corrected.

