# Supplementary material for: Genetic-based patient stratification in Alzheimer’s disease
Source: Sci Rep. 2024 Apr 30;14:9970. doi: 10.1038/s41598-024-60707-1 (PMC11063050; doi:10.1038/s41598-024-60707-1)
Supplement: Supplementary file 1 — Supplementary Information. [file 41598_2024_60707_MOESM1_ESM.pdf]

Supplementary Information

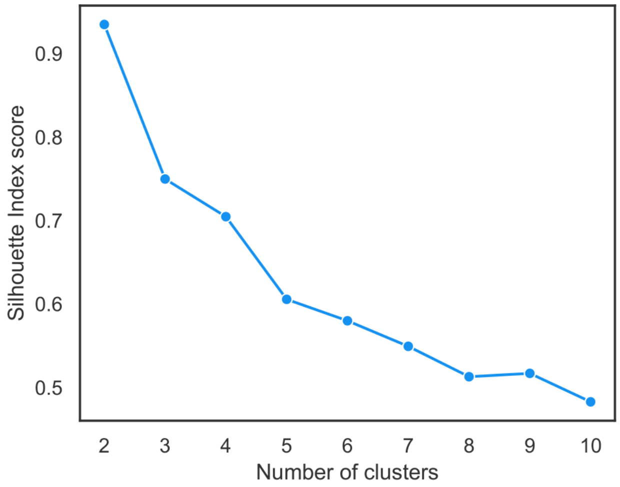

Supplementary Figure 1. SI scores obtained for different numbers of clusters. Clustering model: Similarity Network Fusion and spectral clustering (μ = 0.3 and K = 70 neighbors).

Supplementary Table 1. Significantly different edge scores between clusters. Multiple comparison analysis was computed using ANOVA, corrected with a post-hoc Tukey HSD test. Cluster 1, Cluster 2, and Cluster 3 columns show the mean edge weight in each cluster.

Edge	Cluster 1	Cluster 2	Cluster 3	F-stat	p-value	Signif.	Tukey
APOE-APP	0.5546	0.5819	0.4845	9.47	8.70E-05	***	a, b, c
NSF-APP	0.5411	0.6979	0.4757	747.11	7.50E-177	***	a, b, c
DRD2-NSF	0.4359	0.6333	0.5211	223.10	3.12E-76	***	a, b, c
VCP-NSF	0.3061	0.5611	0.4539	408.13	1.85E-119	***	a, b, c
UCHL1-APP	0.7255	0.7393	0.5117	197.36	3.71E-69	***	a, b, c
MAPT-APOE	0.3962	0.7840	0.6711	939.17	5.53E-202	***	a, b, c
MAPT-PIN1	0.3754	0.8300	0.6829	1055.65	1.78E-215	***	a, b, c
MAPT-PRKN	0.4219	0.8536	0.7086	1030.53	1.16E-212	***	a, b, c
MAPT-APP	0.4351	0.8394	0.6614	1066.65	1.08E-216	***	a, b, c
MAPT-LRRK2	0.4817	0.8239	0.7105	803.48	1.18E-184	***	a, b, c
MAPT-S100B	0.3705	0.8045	0.6529	1043.82	3.70E-214	***	a, b, c
MAPT-MARK4	0.5318	0.7909	0.7233	456.85	4.13E-129	***	a, b, c
PSEN1-MAPT	0.4220	0.8463	0.7105	1040.91	7.86E-214	***	a, b, c

APP-LPL	0.7009	0.7077	0.5405	40.74	1.71E-17	***	b, c
APP-SOD2	0.6662	0.6701	0.4298	145.68	6.58E-54	***	b, c
APP-LRP1	0.7312	0.7429	0.5000	241.53	4.14E-81	***	b, c
APP-PRNP	0.7018	0.7074	0.4532	182.65	5.77E-65	***	b, c
APP-CTSD	0.7578	0.7617	0.5322	337.88	2.07E-104	***	b, c
APP-MAPK7	0.7495	0.7519	0.4868	1052.27	4.22E-215	***	b, c
APP-TGFB1	0.6477	0.6428	0.4181	138.82	9.07E-52	***	b, c
APP-PRKN	0.7389	0.7372	0.5263	126.45	7.77E-48	***	b, c
APP-GYS1	0.7484	0.7505	0.4868	954.92	7.07E-204	***	b, c
APEX1-APP	0.6757	0.6791	0.4298	150.44	2.24E-55	***	b, c
HTRA2-APP	0.7457	0.7451	0.4803	666.44	5.73E-165	***	b, c
KLC1-APP	0.5754	0.5776	0.3900	129.67	7.20E-49	***	b, c
NOS3-APP	0.6216	0.6257	0.4006	152.51	5.20E-56	***	b, c
PIN1-APP	0.6030	0.6065	0.3713	185.11	1.13E-65	***	b, c
AGER-APP	0.7707	0.7707	0.5380	510.08	5.09E-139	***	b, c
PSEN2-APP	0.6567	0.6506	0.4298	129.79	6.61E-49	***	b, c
PSEN1-APP	0.7130	0.7208	0.5482	80.40	2.92E-32	***	b, c
A2M-APP	0.7085	0.7115	0.4415	191.52	1.67E-67	***	b, c
APOE-LRP1	0.5014	0.5457	0.5789	4.44	1.21E-02	*	a
LRP8-APOE	0.4915	0.5287	0.5526	3.84	2.19E-02	*	a
PSEN1-APOE	0.5328	0.5674	0.6018	4.33	1.34E-02	*	a
MC1R-MAPK7	0.8162	0.7931	0.8860	3.78	2.33E-02	*	c
A2M-LRP1	0.7415	0.7648	0.6711	3.88	2.12E-02	*	c

Significant codes: * p-value < 0.05; *** p-value < 0.001
Tukey HSD codes: a Cluster 1 vs. Cluster 2; b Cluster 1 vs. Cluster 3; c Cluster 2 vs. Cluster 3

Supplementary Table 2. Distribution of patients considered for the hypometabolism neuroimaging analysis. Distributions are shown as: number of subjects (neuroimaging observations during time). MCI: Mild Cognitive Impairment

Variable	Cluster 1	Cluster 2	Cluster 3	Controls
N (observations)	209 (530)	425 (1063)	33 (94)	301 (297)
MCI / Dementia *	128 / 13	260 / 31	15 / 2	*NA*

* Note that not all MCI and Dementia subjects described at baseline in each cluster were present because they did not have FDG-PET neuroimaging available, and the diagnosis distributions only differed slightly in the case of Dementia subjects because we queried the diagnosis closest to the neuroimaging dates.

Supplementary Table 3. (Excel file) Significant ROIs found at **baseline** analysis. ROIs names abbreviations are based on AAL 3 atlas publication [S1], and regions categorization is based on the work by [S2]. q-values stand for FDR corrected p-values.

Supplementary Table 4. (Excel file) Significant ROIs found at **longitudinal** analysis. ROIs names abbreviations are based on AAL 3 atlas publication [S1], and regions categorization is based on the work by [S1]. q-values stand for FDR corrected p-values.

Supplementary Table 5. Global graph metrics of clusters' cognitive networks. Multiple comparison analysis was computed using ANOVA, corrected with a post-hoc Tukey HSD test. Cluster 1, Cluster 2, and Cluster 3 columns show the mean and standard deviation values of each metric. Metrics values were obtained through bootstrap calculation (n = 10, 250 repeats).

Graph metric	Cluster 1	Cluster 2	Cluster 3	F-stat	p-value	Signf.	Tukey
Diameter	1.82 ± 0.38	1.9 ± 0.29	2.0 ± 0.0	25.85	0.00	***	a, b, c
Density	0.5 ± 0.14	0.48 ± 0.14	0.43 ± 0.09	24.12	0.00	***	b, c
Average degree	6.05 ± 1.73	5.77 ± 1.66	5.13 ± 1.12	24.12	0.00	***	b, c
Average CC	0.49 ± 0.16	0.47 ± 0.16	0.4 ± 0.11	25.81	0.00	***	b, c

Supplementary Table 6. ADAS-Cog items description.

Code	Description	Cognitive domain	Score range
Q1_WR	Word Recall	Memory	0-10
Q2_Comm	Commands	Language	0-5
Q3_CP	Constructional Praxis	Visuospatial	0-5
Q4_DWR	Delayed Word Recall	Memory	0-10
Q5_NT	Naming Task	Language	0-5
Q6_IP	Ideational Praxis	Visuospatial	0-5
Q7_O	Orientation	Orientation	0-8
Q8_WRT	Word Recognition Task	Memory	0-12
Q9_RTI	Remembering Test Instructions	Memory	0-5
Q10_Comp	Comprehension	Language	0-5
Q11_WFD	Word Finding Difficulty	Language	0-5
Q12_SL	Spoken Language ability	Language	0-5
Q13_NC	Number Cancellation	Attention	0-5

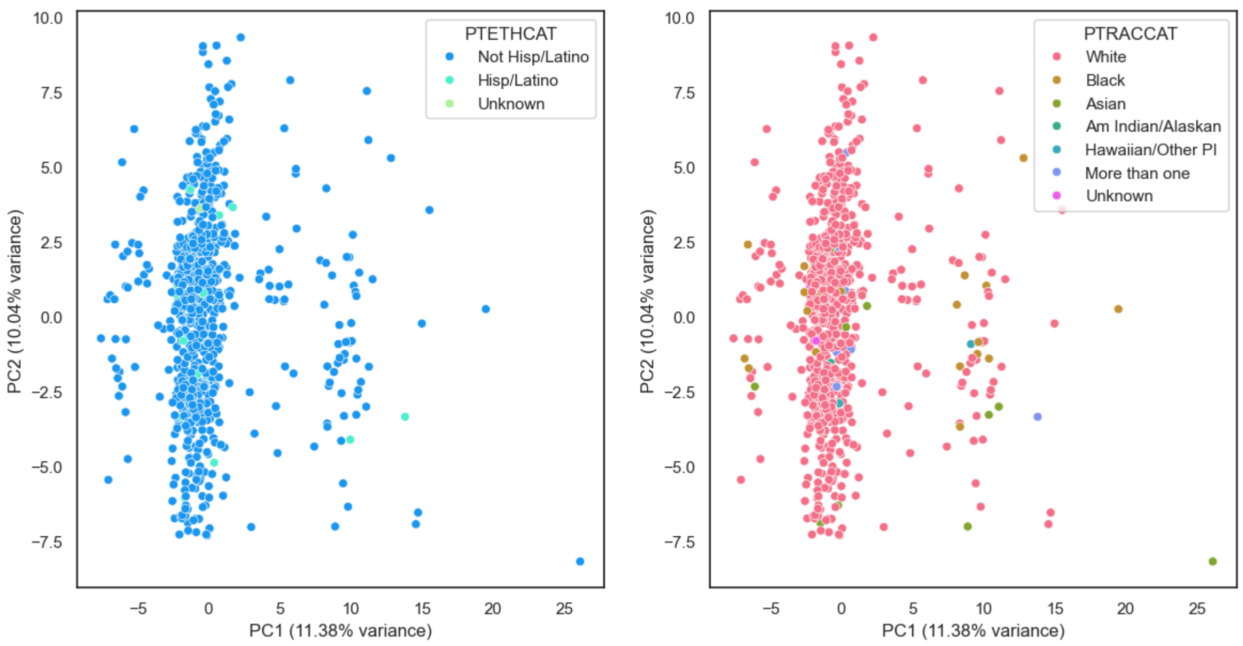

Supplementary Figure 2. Principal Components Analysis on edge scores data **before** subjects filtering by "PTRACCAT" ("Racial categories") and "PTETHCAT" ("Ethnic Category") variables from ADNIMERGE.

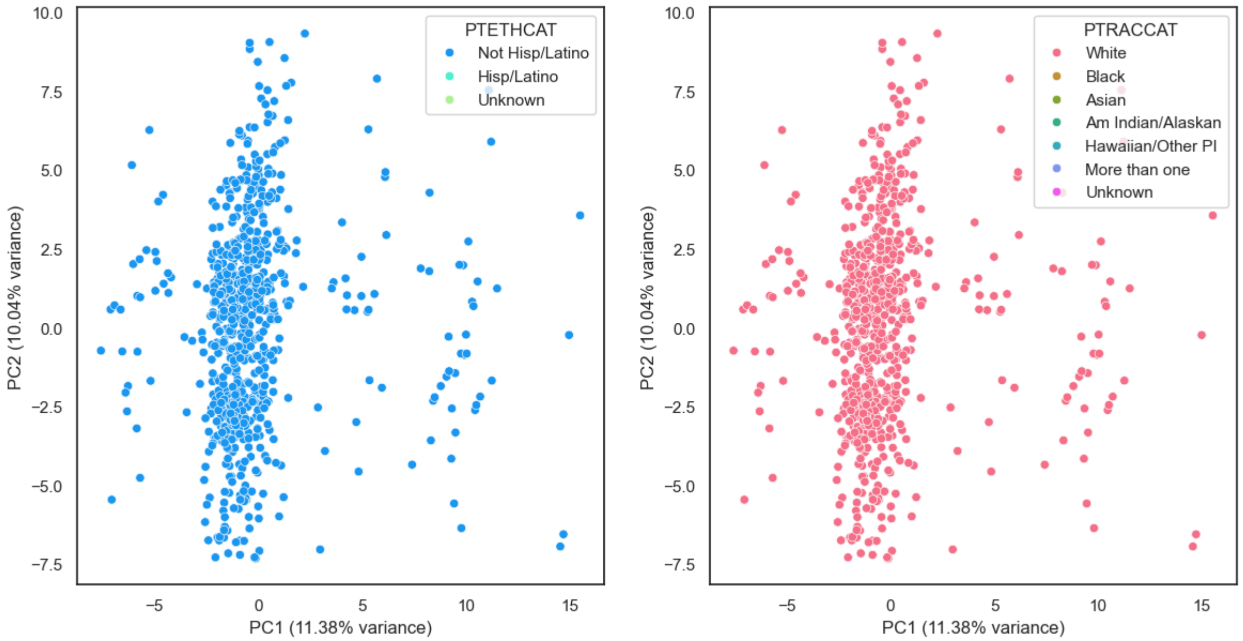

Supplementary Figure 3. Principal Components Analysis on edge scores data **after** subjects filtering by “PTRACCAT” (“Racial categories”) and “PTETHCAT” (“Ethnic Category”) variables from ADNIMERGE.

References

S1. Rolls, E.T., Huang, C.C., Lin, C.P., Feng, J., and Joliot, M. (2020). Automated anatomical labelling atlas 3. NeuroImage *206*, 116189. 10.1016/J.NEUROIMAGE.2019.116189.

S2. Wang, K., Liang, M., Wang, L., Tian, L., Zhang, X., Li, K., and Jiang, T. (2006). Altered Functional Connectivity in Early Alzheimer’s Disease: A Resting-State fMRI Study. Hum. Brain Mapp. *28*, 967–978. 10.1002/hbm.20324.
